# Early enteral feeding following flap surgery for head and neck defects: a quality improvement project using the Plan-Do-Study-Act cycle

**DOI:** 10.1007/s00520-026-11231-7

**Published:** 2026-09-23

**Authors:** Florence Cook, Amelie Niemann, Gareth Ambler, Axel Sahovaler, Phillip Ward

**Affiliations:** 1 Head and Neck Academic Centre, Division of Surgery and Interventional Science, University College London, Charles Bell House, 43-45 Foley Street, London, UK; 2 Head and Neck Centre, University College London Hospital NHS Foundation Trust, London, NW1 2PG UK; 3 Department of Perioperative Medicine, University College London Hospital NHS Foundation Trust, London, NW1 2PG UK; 4 Department of Statistical Science, University College London, 1-19 Torrington Place, London, WC1E 7HB UK

**Keywords:** Postoperative, Nutrition, Oral feeding, Head and neck cancer, Enhanced recovery after surgery, Flap

## Abstract

**Purpose:**

Patients undergoing reconstructive head and neck (H&N) surgery are often made nil-by-mouth postoperatively, requiring enteral feeding. Early enteral feeding (≤ 24 h) after surgery is recommended as part of enhanced recovery. Limited literature has investigated timing to enteral feeding in H&N.

**Methods:**

A quality improvement project was undertaken at a London network guided by the Plan-Do-Study-Act cycle. Usual care was to wait until the morning review before commencing enteral feeds. A change of approach was queried to bring this forward e.g. same day of surgery. Five consultants agreed to trial earlier enteral nutrition (EEN); two opted to continue with usual care (UEN). Data were collected prospectively on fasting time, nutritional intake and complications. Groups were compared using t-test or Mann–Whitney for continuous data and Fisher’s exact for categorical data.

**Results:**

Forty-five patients were included (*n* = 23 EEN, *n* = 22 UEN) between May and November 2025. The EEN group had significantly lower average preoperative fasting time than UEN group (median [Q1, Q3]: 0.7 [0.6, 0.9] vs 1.3 [1.2, 1.5] days *p* < 0.001), time to commencing EN (median [Q1, Q3]: 4.3 [3.3, 5.7] vs 18.5 [17.4, 19.9] hrs *p* < 0.001) feed in first 24 hrs (215 ml vs 0 ml *p* ≤ 0.001), energy (322.5 kcal vs 0 kcal *p* < 0.001) protein intake (13.5 g vs 0 g *p* < 0.001) and weight loss (median 1% vs 5.6% *p* = 0.02). No statistically significant differences were observed between Clavien-Dindo (grade ≥ 3 35% EEN vs 27% UEN, *p* = 0.92), or length of stay (13 days vs 14.5 days *p* = 0.92).

**Conclusion:**

This project helps demonstrate proof-of-concept of EEN in a naturalistic setting. Patients receiving EEN had reduced fasting times without increased complications. Providing nutrition in the immediate postoperative period may generate additional benefits, which further research should establish.

## Background

Surgery is the mainstay for treating head and neck (H&N) cancer of the oral cavity. Larger tumours that leave "volume defects" can be reconstructed with flap tissue transfer to optimise functional outcomes [1]. Following flap surgery, patients are made nil-by-mouth to allow recovery of wounds and swallow musculature. During this period, enteral nutrition is provided as a safe route of nutrition via a naso-gastric tube or gastrostomy [2].

Enhanced recovery after surgery (ERAS) protocols are multimodal perioperative care pathways that aim to achieve early recovery by optimising preoperative function and reducing stress response after surgery. This includes early mobilisation, standardised analgesic and anaesthetic regimens, and nutritional optimisation which can lead to significant reductions in hospital stay and associated costs. It has been reported that 60% of patients are malnourished at presentation, [3, 4] and poor nutrition is an independent risk factor for surgical complications, morbidity and mortality [5]. To avoid exacerbating malnutrition, a key component of ERAS protocols includes optimising perioperative nutrition. This includes avoiding prolonged fasting by allowing solids up to 6 hrs prior to anaesthesia and fluids including carbohydrate loading up to 2 hrs prior to surgery, and reintroducing nutrition within 24 hrs of surgery.

The ERAS Society H&N Consensus guidelines recommend that postoperative tube feeding should be initiated within 24 hrs of surgery in patients for whom oral feeding cannot be tolerated [2]. The practice of preoperative fasting stems from concerns of pulmonary aspiration after surgery; however, it been found that preoperative fasting can increase hyperglycaemia, metabolic stress and insulin resistance [6]. Previous studies have found that reducing the fasting period by allowing fluids up to 2 h prior to surgery does not increase the risk of aspiration, morbidity or complications. Carbohydrate loading has also been found to attenuate postoperative insulin resistance, reduce protein and nitrogen losses, improve postoperative muscle function and reduce length-of-stay (LoS) [7, 8]. Early postoperative nutrition, and avoiding prolonged nil-by-mouth can enhance the metabolic response, leading to less insulin resistance, nitrogen loss and muscle loss [9]. Few studies have been conducted in H&N investigating efficacy of this approach with the majority conducted in gastrointestinal surgery, concluding no benefit for keeping patients nil-by-mouth. A Cochrane review demonstrated that early enteral nutrition (EN) reduced LoS by almost two days. Whilst findings were inconclusive with regards to benefits for other outcomes (postoperative complications, mortality, adverse events and quality-of-life (QoL)), existing studies have found no differences in complication rates, including pneumonia for patients receiving early versus delayed enteral feeding, and there is weak evidence to support increased risk of vomiting [10, 11]. Most updated recommendations from the ESPEN (European Society of Parenteral and Enteral Nutrition) Society recommends *‘oral/tube feeding shall be started as soon as possible within the first few hours after surgery in conscious and haemodynamic stable patients’* [12]*.* Due to improvements in postoperative care including analgesia and attention to fluid balance, patients may be more able to tolerate early enteral feeding today than when compared to previous years [11].

Few studies have explored timing to initiation of postoperative tube feeding in H&N. This includes adherence to the < 24 hrs guidelines, a trial of intraoperative EN [13], and ERAS protocols stipulating commencement of EN within 12 hrs of surgery [14]. A gap in the literature exists investigating timing to EN within the < 24 hrs period. The aim of this project was to establish whether it is possible to introduce EN earlier, including the same day of surgery for patients undergoing reconstructive H&N surgery.

## Methods

### Setting/context

University College London Hospital (UCLH) is a centralised H&N surgery network covering a wide geographic area across North/Central and East London. An existing ERAS protocol in this unit stipulates that enteral feeding should commence within 24 hrs of surgery, in compliance with international enhanced recovery guidelines [2]. Previous local audits in our department have found adherence rates to EN within 24 hrs of surgery were 94% for major H&N surgery in 2022 [15].

### Intervention

A quality improvement project was proposed to reduce the postoperative fasting time after surgery using the Plan-Do-Study-Act (PDSA) cycle [16]. This comprises a four-stage cyclic approach: Stage One (Planning) where a change aimed at improvement is identified, Two (Do) where the change is tested, Three (Study), where the success of the change is examined and Four (Act) where any adaptations are identified to inform the next steps.

### Plan

Existing ERAS protocols in this unit aim to establish enteral feeding within 24 hrs of surgery, per the ERAS Society recommendations. Following major flap surgery, patients are kept nil-by-mouth / tube until the next morning upon surgical review to confirm that feeding can be commenced. A change in approach was proposed by an OMFS surgeon, who felt that feeding could be established sooner, such as on the same day of surgery and/or before the morning ward round. Patient and Public Involvement feedback also indicated that feeding earlier may support energy levels for mobilising the next day. This was discussed amongst the key stakeholders involved (consultant H&N surgeons, anaesthetists/perioperative medicine staff, nursing and dietetic teams). It was acknowledged that finishing times for surgery were sooner than historically (with midnight finishes uncommon). During these discussions, concerns were also raised that most patients were being sent for chest x-ray (CXR) prior to attempting pH aspirate for NGT placement, leading to unnecessary CXRs and/or delayed feeding. National/local guidance stipulates that position checking of NGT should be via pH aspirate in the first instance before deferring to a CXR [17].

### Do

A draft protocol was developed by the H&N Dietitian (FC), OMFS and Perioperative Medicine Consultant (AS, PW). This was circulated to the key stakeholders and subsequently underwent three rounds of revision (April to May 2025) which included:

1. Agreement on which OMFS consultants and their respective cases could be for EEN: Five OMFS consultants were in agreement to trialling earlier EN (EEN) and two OMFS consultants were not. The main rationale against a change in approach was overall risk versus benefit ratio and safety concerns of nausea/vomiting.
2. Agreement on patient eligibility for EEN: high-risk patients included those identified as high risk of vomiting or delayed gastric emptying preoperatively, to be excluded from EEN per operating consultant discretion.
3. Risk management of any potential return to theatres (RTT): No changes followed as the perioperative medicine team advised they could risk manage any feed in patients.

**Fig. 1:**
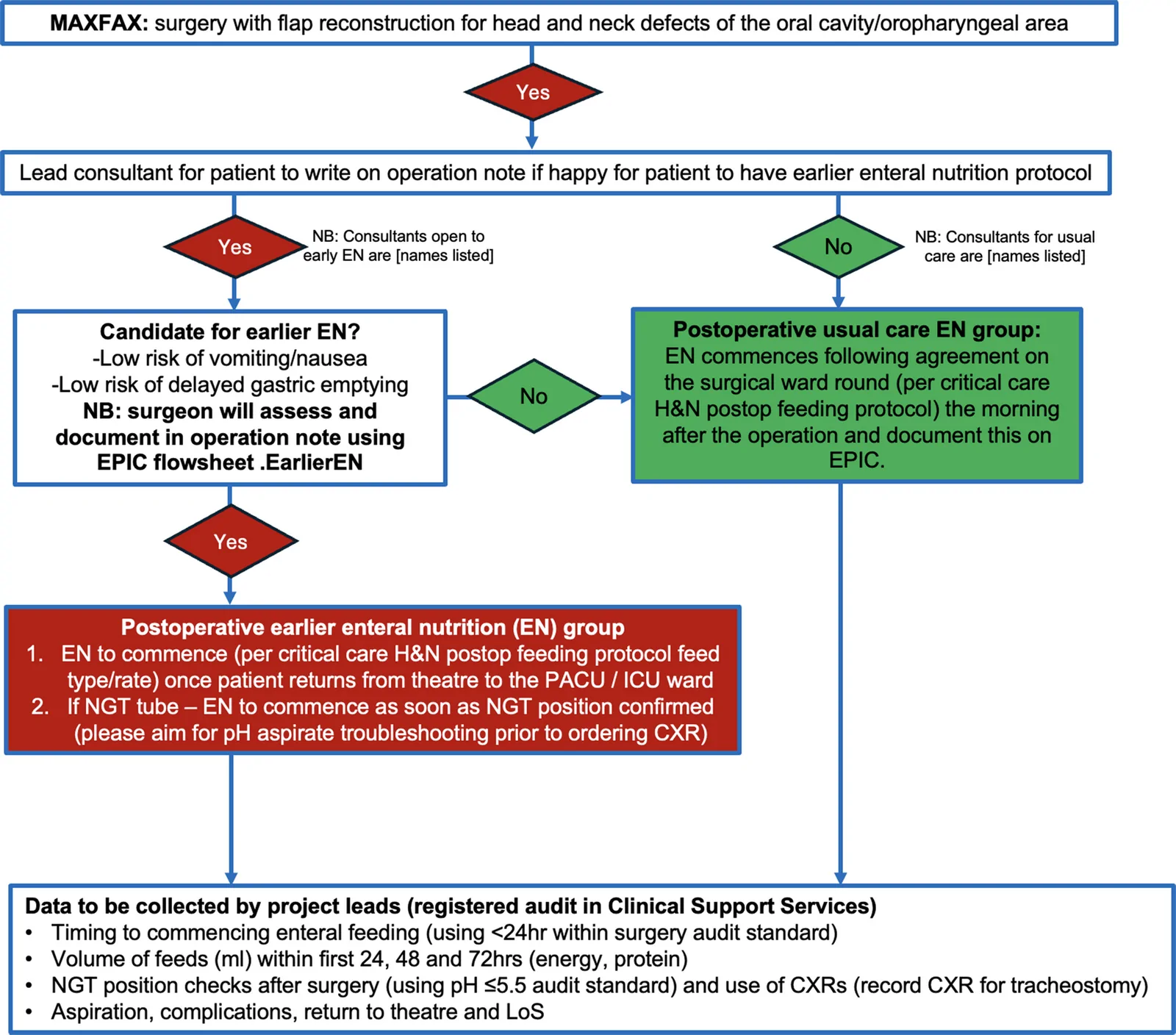
Postoperative feeding protocol for H&N Surgery QI Project. Abbreviations: CXR = chest x-ray, EN = enteral nutrition, PACU = Post-anaesthesia care unit, ICU = intensive care unit, NGT = naso-gastric tube. EPIC = software used for electronic medical record), LoS = length of stay

The finalised protocol (Fig. 1) was agreed and then approved through the H&N clinical governance meeting before being rolled out on 27th May 2025. An electronic record ‘smartphrase’ (a pre-written template that can be added to patient notes) was built to ensure postoperative feeding instructions were clear and consistent. It was agreed that the protocol would be trialled for six months, with checkpoints at one and three months. The type of enteral feed and rate was not changed and followed the established starter enteral feeding regime for H&N surgery. This comprised a 1.5 kcal/ml standard polymeric feed commencing at a slow rate of 20 ml/hr and increasing by 30 ml/hr if aspirates are < 200 ml with target feed rate dependent on the patient’s weight (ranges from 35 to 72 ml/hr over 24 hrs, reducing to 20 hrs once the patient is 48 hrs after surgery).

### Study

Data were collected prospectively for six months at the point of introducing the new protocol using audit standards from the ERAS H&N Consensus guidelines:

*‘In patients for whom oral feeding cannot be established postoperative tube feeding should be initiated within 24 hours’.*

### Sample

#### Inclusion criteria

Adults (≥ 18 years) undergoing reconstructive flap surgery for H&N defects of the oral cavity and oropharynx. This included cancer, ORN, benign disease and secondary reconstruction commencing enteral nutrition after surgery.

#### Exclusion criteria

- Children/adolescents < 18 years
- ENT cases (e.g. laryngectomies)
- Patients who did not commence enteral nutrition after surgery or contraindicated (e.g. commenced oral feeding or parenteral nutrition)

### Data collection

The primary outcome was to identify timing to commencing enteral feeding after surgery and perioperative fasting time. Data were also collected on surgical/demographic factors, type of surgery (including presence of tracheostomy), type of feeding tube, volume of enteral feed including energy (kcal) and protein (g) provided within the first 24 hrs of surgery (6am day of surgery to 6am the day after surgery), LoS and complications including surgical (e.g. wound breakdown), aspirates and incidence of nausea/vomiting. Clavien-Dindo score and weight were collated on discharge (or up to 30 days postoperative if admission surpassed 30 days).

### Analysis

Data were collated using Microsoft Excel (Microsoft Office, version 16.103.2) and analysed using SPSS (IBM version 29) and Stata (StataNow MP 18.5). Distribution of data was assessed and presented as mean (SD) for normally distributed data, or median [Q1, Q3] for skewed/ordinal data. Between group differences for earlier (EEN) versus usual enteral nutrition (UEN) were compared for timing to enteral feeding, volume of feed, energy and protein intake and complications using unpaired student *t*-test or Mann–Whitney *U* test. Categorical variables were presented as frequencies (%). Fisher’s exact test was used to compare differences for presence of complications including vomiting and wound breakdown/dehiscence.

### Act

Findings were presented to the wider H&N surgical team at an audit meeting in December 2025, to determine whether EEN could be established as part of the H&N ERAS protocol.

### Ethical considerations

This project was registered as audit/quality improvement in the clinical support services department (Audit number CSS-64). Ethical approval was not required in accordance with the Health Research Authority decision tool (https://www.hra-decisiontools.org.uk/research/) as this was considered service improvement (improving timing to feeding within the accepted standard of aiming to feed within 24 h of surgery) and audit (for data collection against the 24 h standard). Data protection regulations were adhered to: patients who had signed for NHS data opt-out were excluded.

Findings were reported in accordance with the Standards for Quality Improvement Reporting Excellence 2.0 (SQUIRE) guidelines.

## Results

Forty-eight patients had flap surgery who were eligible for inclusion. Three patients were excluded due to NHS data opt-out. 45 patients were included (*n* = 23 EEN and *n* = 22 UEN). Six patients who were recommended EEN were not given this (and were subsequently included in the UEN group for analysis) due to the following reasons: had immediate RTT before feed had commenced (*n* = 2), were awaiting CXR and did not receive EN until after the morning ward round (*n* = 2), displaced NGT and required replacement (*n* = 1), no reason given (*n* = 1).

Demographic information is illustrated in Table 1. The EEN and UEN groups were broadly similar; however, the UEN group had a higher number of sarcomas (22% vs 0%).

**Table 1:** Demographic and surgical characteristics of early and usual enteral feeding groups

Demographics	EEN (*n* = 23) *N*, %	UEN (*n* = 22) *N*, %
Age (years) Mean (SD) Sex (male, female)	58 (17.7) 12 (52.2),11 (47.8)	53 (17.0) 13 (59.1), 9 (40.9)
**Diagnosis**		
SCC AJCC Stage 1–2 SCC AJCC Stage 3–4 Recurrent disease Benign or dysplasia Osteoradionecrosis Sarcoma Other	6 (26.1) 10 (43.5) 3 (13.0) 1 (4.3) 2 (8.7) 0 (0) 1 (4.3)	3 (13.6) 11 (50) 0 (0) 1 (9.1) 1 (9.1) 5 (21.7) 1 (4.5)
**Defect site**		
Tongue Buccal mucosa Mandible/soft palate/RMT Maxilla/hard palate FOM Lip Other	4 (17.4) 5 (21.7) 7 (30.4) 0 (0) 4 (17.4) 1 (4.3) 2 (8.7)	4 (18.2) 2 (9.1) 6 (27.3) 5 (22.7) 3 (13.6) 1 (4.5) 1 (4.5)
**Surgery**		
Neck dissection	20 (87.0)	21 (95.5)
Free flap	21 (91.3)	20 (90.9)
Pedicled or local flap	2 (8.7)	2 (9.1)
Tracheostomy	15 (65.2)	17 (77.3)
**Preop CHO loading**		
Yes Contraindicated Not given	17 (73.9)* 5 (21.7) 1 (4.3)	16 (72.7)** 4 (18.2) 2 (9.1)

All values are *N* (%) unless otherwise indicated. *n = 2 pre-diabetes **n = 1 pre-diabetes. Abbreviations: *SCC*, squamous cell carcinoma; *RMT*, retromolar trigone; *FOM*, floor of mouth; *AJCC*, American Joint Committee on Cancer Staging—8th Edition (pathological stage); *CHO*, carbohydrate; *EEN*, earlier enteral nutrition (before next day surgical review); *UEN*, usual enteral nutrition (after next day surgical review)

Timing to feeding and complication data is illustrated in Table 2. Overall, 96% (*n* = 33) patients received EN within 24 hrs of surgery. Only 2 patients did not meet this target due to immediate RTT. The EEN group had significantly shorter perioperative fasting time (0.7 vs 1.3 days, *p* < 0.001) and time to commencing EN (4.3 vs 18.5 hrs, *p* < 0.001). In addition, the EEN group received significantly higher volume of feed (215 ml vs 0 ml, *p* < 0.001) in the first 24 hrs from being fasted for surgery and respective energy (322.5 kcal vs 0 kcal, *p* < 0.001) and protein (13.5 g vs 0 g, *p* < 0.001) intake. There was no significant difference in complications (Clavien-Dindo scores, *p* = 0.92), vomiting in the first 24 (*p* = 0.61) or 72 hrs (*p* = 1.00) or aspirates in the first 72 hrs (*p* = 0.56). The UEN group had longer LoS (14.5 vs 13 days, *p* = 0.92) and ICU LoS (5 vs 3 days, *p* = 0.06) though these were not significant.

**Table 2:** Feeding and complication characteristics of patients undergoing early or usual enteral feeding

Characteristics	EEN (*n* = 23)	UEN (*n* = 22)	*p* value
Perioperative fasted time (days)*			
Median [Q1, Q3]	0.7 [0.6, 0.9]*	1.3 [1.2, 1.5]*	< 0.001
Time to commencing EN (hrs)			
Median [Q1, Q3] Same day of surgery ≤ 12:00 a.m. Next day of surgery > 12:00 a.m.	4.3 [3.3, 5.7]* 22 (96%) 1 (4%)^a^	18.5 [17.4, 19.9]* 0 (0%) 22 (100%)	< 0.001
Volume of feed (ml)** Median [Q1, Q3] Energy intake (24 h) (kcal) Median [Q1, Q3] Protein intake (24 h) (g) Median [Q1, Q3]	215 [140, 340]* 322.5 [210, 510]* 13.5 [8.8, 21.3]*	0 [0, 0]* 0 [0, 0]* 0 [0, 0]*	< 0.001 < 0.001 < 0.001
Initial pH taken (*n* = 43)^b^ CXR (total during admission, Median Q1, Q3) Total cost of CXRs (£50/CXR)	4/22 (18.2%) 53; 2 [1, 3] £2650	3/21 (14.3%) 73; 3 [2, 4] £3650	
**Complications**			
Wound breakdown/dehiscence	7 (30.4%)	8 (36.4%)	0.76
Flap failure	0 (0%)	1 (4.5%)	1.00
RTT within 24 hrs	2 (8.7%)	2 (0.1%)	
Clavien-Dindo score Median [Q1, Q3]	2 [2, 3]	2 [2, 3.5]	0.92
0 1 2 3a 3b	1 (4.3%) 3 (13.0%) 11 (47.8%) 4 (17.4%) 4 (17.4%)	0 (0%) 3 (13.6%) 13 (59.1%) 0 (0%) 6 (27.3%)	
**Vomiting**			
First 24 hrs, 72 hrs	1 (4.3%), 2 (8.7%)	2 (9.1%), 3 (13.6%)	0.61
Aspirates (first 72 hrs) Median [Q1, Q3]	40 [8.5, 241]	47.5 [6.8, 110.8]	0.56
**Prokinetics/antiemetics**			
Yes Prescribed, not given***	16 (69.6%) 7/23 (30.4%)	16 (76.2%) 5/21 (23.8%)	
ICU LoS median [Q1, Q3] LoS median [Q1, Q3]	3 [2, 5] 13 [10.5, 19.5]	5 [3, 9] 14.5 [9.5, 17.5]	0.06 0.92
% weight change Median [Q1, Q3] Duration of weight change (days) Median [Q1, Q3]	1 [0.8, 4] 12 [10, 14]****	5.6 [3, 9] 17 [10, 22]	0.02

All values are *N* (%) unless otherwise indicated. Data presented as Median [Q1, Q3] for skewed data or Mean (SD) for normally distributed data or *n* (%) for categorical data. *Perioperative fasting time was defined as time until EN was commenced from last food/drink e.g. carbohydrate loading or last meal e.g. night before surgery. **Volume of feed was calculated from 6am day of surgery (at the point of being fasted) to 6am day after surgery. ***for *n* = 44 ****for *n* = 22. EEN, earlier enteral nutrition (before next day surgical review); *UEN*, usual enteral nutrition (after next day surgical review) a *n* = 1 commenced feed at 3am as CXR not reported until 2am. b *n* = 2 participants had gastrostomy

Sixteen percent (*n* = 7/43) of patients with NGT insitu (remaining *n* = 2 had gastrostomy) had position checked with pH aspirate before deferring to CXR. Fewer CXRs were undertaken in the EEN group during their admission (53 vs 73 CXRs).

## Discussion

This project aimed to assess if it is possible to introduce EEN in a naturalistic setting, using the PDSA QI cycle. In the prospective audit, we found that patients who received EEN had reduced perioperative fasting time, reduced timing to commencing EN after surgery and improved nutrition intake in the first 24 hrs from being fasted for surgery, compared to those with UEN. In addition, we observed no difference in complication rates during admission, vomiting or aspirates in the first 24 and 72 hrs.

The majority (96%) of patients in this project met the ERAS target of feeding within 24 hrs, demonstrating this is broadly feasible in practice. Our adherence rate is higher than previous studies; Imai and colleagues (2024) found that EN could be started in 79.4% and 97.1% of patients within 24 and 48 hrs respectively, with free jejunal transfer observed as an independent risk factor for not being able to commence early EN [18].

The benefits of EEN observed in this project also align with existing studies. Yamamoto and colleagues (2024) conducted a retrospective before and after study, where patients who received EN on postoperative day one (early) were compared to those that received EN on postoperative day two, following physician review. Authors found that early EN was safe, feasible and did not increase complications [19]. Recently, a randomised controlled trial has investigated whether intraoperative EN is feasible. Authors reported no significant differences in adverse events or flap failure, whilst enhancing wound regeneration, indicating safety of this intervention [13].

### Strength and limitations

The strengths of this project include the structured approach to quality improvement using the PDSA cycle which is widely accepted model in healthcare [20]. Limitations include the small sample size, short timeframe of the project and non-randomised approach which increases risk of bias and means causation cannot be established. In addition, the single-centre design limits the generalisability of our findings. However, in the context of QI, we hope that findings help indicate proof-of-concept that EEN is safe and possible in a naturalistic setting and this may be transferable to other H&N surgical units offering flap surgery.

### Further research

In the final stage of the PDSA cycle (Act), findings were presented to the H&N surgical team. In view of no difference in complication rates between the early and usual feeding groups and superior outcomes for nutritional intake and reducing perioperative fasting time in the EEN group, it was agreed that EEN could be established as an option in the local ERAS protocol. The high use of CXRs used to confirm NGT position without attempting pH aspirate first was highlighted to the senior staff on the PACU unit, and further PDSA cycles are planned to focus on reducing the use of unnecessary CXRs.

Future high-quality randomised trials should investigate EEN versus UEN in H&N surgery to confirm safety, superiority and/or non-inferiority of EEN. In addition, it is likely there are additional metabolic benefits of EEN which future studies should investigate. A mapping exercise investigating current practices for timing of EN after H&N surgery may also be necessary, as it has also been acknowledged that future trials in gastrointestinal surgery may be challenging due to the fast-track protocols becoming usual care [11].

## Conclusion

This QI project suggests that EEN (before surgical review the next day and as early as the same day of surgery) was safe and did not lead to increased complications compared to UEN (feeding post-surgical review the next day) in a naturalistic setting. Benefits of EEN include reduced perioperative fasting times and higher energy/protein provided in the first 24 hrs of surgery.

## Data Availability

The data that support the findings of this study are not openly available due to reasons of sensitivity and are available from the corresponding author upon reasonable request.
